# Mapping immune checkpoint inhibitor side effects to item libraries for use in real-time side effect monitoring systems

**DOI:** 10.1186/s41687-025-00855-8

**Published:** 2025-03-06

**Authors:** Julia Lai-Kwon, Michael Jefford, Stephanie Best, Iris Zhang, David Cella, Claire Piccinin, Bryce B. Reeve, Claudia Rutherford

**Affiliations:** 1 Department of Medical Oncology, Peter MacCallum Cancer Centre, Melbourne, Australia; 2 Department of Health Services Research, Peter MacCallum Cancer Centre, Melbourne, Australia; 3 Australian Cancer Survivorship Centre, Peter MacCallum Cancer Centre, Melbourne, Australia; 4 Sir Peter MacCallum Department of Oncology, University of Melbourne, Melbourne, Australia; 5 Victorian Comprehensive Cancer Centre Alliance, Melbourne, Australia; 6 Australian Genomics, Murdoch Children’s Research Institute, Melbourne, Australia; 7 Department of Medical Social Sciences and Robert H. Lurie Comprehensive Cancer Centre, Northwestern, Chicago, IL USA; 8 Quality of Life Department, European Organisation for Research and Treatment of Cancer (EORTC), Brussels, Belgium; 9 Department of Population Health Sciences, Duke University School of Medicine, Durham, NC USA; 10 Sydney Quality of Life Office, Susan Wakil School of Nursing and Midwifery, Faculty of Medicine and Health, The University of Sydney, Sydney, Australia; 11 The Daffodil Centre, a Joint Venture with Cancer Council New South Wales, The University of Sydney, Sydney, Australia

**Keywords:** Symptom monitoring, Patient-reported outcomes, Digital health, Immunotherapy, Immune checkpoint inhibitors, Item libraries, Item lists

## Abstract

**Background:**

Monitoring for the side effects of novel therapies using patient-reported outcomes (PROs) is critical for ensuring patient safety. Existing static patient-reported outcome measures may not provide adequate coverage of novel side effects. Item libraries provide a flexible approach to monitoring for side effects using customized item lists, but the ideal process for matching side effects to items sourced from multiple item libraries is yet to be established. We sought to develop a pragmatic process for mapping side effects to items from three major item libraries using immune checkpoint inhibitor (ICI) side effects as an example.

**Methods:**

Using a consumer- and clinician-driven list of 36 ICI side effects, two authors independently mapped side effects to Common Terminology Criteria for Adverse Event (CTCAE) terms, and then to three item libraries: the Patient-Reported Outcome version of the Common Terminology Criteria for Adverse Events (PRO-CTCAE), the European Organisation for Research and Treatment of Cancer (EORTC) Item Library, and the Functional Assessment of Chronic Illness Therapy (FACIT) searchable library. The rates of inter-rater agreement were recorded. Following item collation from the item libraries, we devised criteria for selecting the optimal item for each side effect for inclusion in a future electronic PRO system based on guidance from the above groups.

**Results:**

All 36 side effects mapped to at least one CTCAE term, with eight mapping to more than one term. Twenty-three side effects mapped to at least one PRO-CTCAE term, 35 side effects mapped to at least one EORTC item, and 31 side effects mapped to at least one FACIT item. The inter-rater agreement rate was 100% (PRO-CTCAE), 83% (EORTC) and 75% (FACIT). Pre-determined criteria were applied to select the optimal item for each side effect from the three item libraries, producing a final 61-item list.

**Conclusion:**

Using ICI side effects as an example, we developed a pragmatic approach to creating customized item lists from three major item libraries to monitor for side effects of novel therapies in routine care. This process highlighted the challenges of using item libraries and priorities for future work to improve their usability.

**Supplementary Information:**

The online version contains supplementary material available at 10.1186/s41687-025-00855-8.

## Background

Electronic patient-reported outcome (ePRO) symptom monitoring between clinic visits is an evidence-based method of monitoring for cancer symptoms and treatment-related side effects [1–19]. While many ePRO symptom monitoring systems monitor for general cancer or chemotherapy-related symptoms (such as nausea or fatigue), there has been growing interest in monitoring for specific treatment-related side effects, such as from immune checkpoint inhibitors (ICI) [20] or chimeric antigen receptor T cells (CAR-T) [21, 22]. Supporting patients and carers to self-monitor for side effects and self-manage mild side effects might help improve the safe delivery of these therapies.

Patient-reported outcome measures (PROMs) specific to the toxicities of novel therapies are often not available. Existing PROMs may lack content validity for use in routine clinical care. An alternative approach is to create customized item lists [23] generated from item libraries such as the Patient-Reported Outcome version of the Common Terminology Criteria for Adverse Events (PRO-CTCAE) [24–26], the European Organisation for Research and Treatment of Cancer (EORTC) Item Library [27], or the Functional Assessment of Chronic Illness Therapy (FACIT) searchable library [28].

Selected items need to be relevant and consistently understood to ensure that patients accurately report their side effects with minimal burden. Item libraries, which have undergone rigorous development and validation with patients, provide a validated source of items that can be selected to create customized item lists for ePRO monitoring. Several groups have described their process of matching side effects to items from the PRO-CTCAE [29–33], EORTC Item Library [34] and the FACIT searchable library [35]. However, the ideal process of matching side effects to items across multiple item libraries is yet to be established.

We are co-designing an ePRO symptom monitoring system for people receiving ICI for use in routine clinical care [36]. This system will enable people to report potential ICI side effects in real-time and facilitate earlier healthcare professional intervention. As part of this process, we have conducted and reported on a national modified Delphi survey to determine which ICI side effects require monitoring in routine care [37]. We sought to develop a pragmatic process for mapping side effects to items from multiple item libraries, using the example of ICI side effects to detail our process for doing so. The final item list would be included in an ePRO symptom monitoring system for ICI toxicities for use in routine clinical care.

## Methods

### ICI side effects

A consumer- and clinician-driven prioritized list of 36 symptomatic ICI side effects for inclusion in symptom monitoring systems was established through an Australian modified Delphi study [37]. The methodology for this two-round, online, modified Delphi study is described here in brief. The study involved 114 participants, including 56 patients and carers who had received or were receiving ICI and 58 managing healthcare professionals. In Round 1, participants rated the importance of including 63 side effects in an ePRO system for ICI side effects. Side effects rated as ‘important’ or ‘very important’ by > 75% of participants were included in Round 2. Side effects rated as ‘important’ or ‘very important’ by < 50% of participants were excluded. Side effects rated as ‘important’ or ‘very important’ by 50–75% of participants were discussed at a roundtable to determine inclusion in Round 2. Details regarding the conduct of the roundtable are described in Lai-Kwon et al. [37]. Side effects which did not achieve mutual agreement were included in Round 2. In Round 2, participants ranked the 10 most important side effects from a list of 36 side effects generated from round 1. All 36 side effects were included in the final prioritized list.

#### Mapping of side effects to CTCAE terms

The CTCAE (version 5.0) is a descriptive terminology catalogue comprising 838 adverse events developed and maintained by the US National Cancer Institute [38]. It includes symptomatic adverse events, observable adverse events, laboratory and radiographic findings, and diagnoses. The CTCAE can be used by clinicians to grade adverse events in clinical trials and routine care. A grading scale is provided for each side effect from Grade 1 (mild) to Grade 5 (resulting in death).

Each of the 36 side effects identified in the Australian modified Delphi survey was mapped to a corresponding CTCAE version 5.0 term. The rationale for undertaking this process was that the 36 side effects were not originally described in terms of the CTCAE as these were drawn from clinical practice guidelines as described in Lai-Kwon et al. [37]. As the EORTC Item Library and FACIT searchable library are organized according to CTCAE terms, identifying any relevant CTCAE term(s) for each side effect would aid the subsequent mapping process.

Side effect terms that consisted of multiple side effects were split and considered as individual side effects (e.g. ‘eye problems’ was split into ‘protrusion of the eye’, ‘sensitivity of the eyes to light’, ‘eye pain’, and ‘red eye’). Where a side effect did not map exactly to a CTCAE term, the most similar CTCAE term was selected by a multidisciplinary research team including clinicians, patients, and PRO methodologists, by consensus. If there was more than one appropriate CTCAE term for a side effect, all relevant CTCAE terms were included.

Mapping of side effects to the CTCAE was conducted by JLK and IZ independently. Mapping results were then compared to calculate an inter-rater agreement rate.

#### Mapping side effects/CTCAE terms to items within three item libraries

##### PRO-CTCAE

The adult version of the PRO-CTCAE is a library of 124 items measuring 78 symptomatic adverse events available from the US National Cancer Institute [24–26]. For each adverse event, up to three items are administered to evaluate the side effect’s presence, frequency, severity, and/or interference with activities of daily living. Customized item lists consisting of PRO-CTCAE items that reflect anticipated symptomatic adverse events can be developed. For each symptomatic adverse event selected, all relevant symptom attribute items (i.e. presence, frequency, severity, interference) should be administered.

We mapped the 36 ICI side effects and their associated CTCAE term(s) to the PRO-CTCAE using the following process:

1. We searched for each ICI side effect and its CTCAE term(s) using the search function in the PRO-CTCAE Portable Document Format (PDF) form. If a side effect or its CTCAE term(s) did not map directly to a PRO-CTCAE symptom term, the most similar PRO-CTCAE symptom term was selected by JLK and IZ by consensus.
2. Questions relating to the presence, frequency, severity, and/or interference with activities of daily living for that PRO-CTCAE symptom term were recorded. For each question, the recall period and response scale were also recorded.

##### EORTC item library

The EORTC Item Library is an online interactive catalogue currently comprised of over 1000 items from the EORTC Quality of Life Group’s portfolio of 70 questionnaires [27, 39, 40]. It allows more flexible use of the static questionnaires by enabling users to select specific items to create customized item lists [41]. Items are classified according to the CTCAE framework [41] as well as a general EORTC taxonomy to facilitate users finding items using more general terms and a common clinical language. In general, customized item lists generated from the Item Library should supplement the EORTC QLQ-C30 and, where relevant, a disease or treatment-specific module when used for research [40, 42]. However, there is no guidance regarding how the Item Library should be used in conjunction with the EORTC QLQ-C30 for the purposes of ePRO symptom monitoring.

We mapped the 36 ICI side effects and their associated CTCAE term(s) to the EORTC Item Library using the following process:

1. Using the ‘item classification’ by ‘CTCAE classification’ section of the EORTC Item Library, we searched for each of the 36 ICI side effects and its CTCAE term(s).
2. For each corresponding Item Library item, the Item Library code and source questionnaire(s) were recorded.
3. All identified Item Library items were reviewed, and duplicates removed.

##### FACIT searchable library

The FACIT measurement system includes over 100 self-report questionnaires assessing symptoms, functional abilities and general perceptions of health and well-being as well as other aspects of health-related quality of life (HRQL). The FACIT searchable library comprises over 700 items from these questionnaires for use in adults aged 18 and over, enabling the creation of customized item lists [28].

Using the FACIT searchable library [28], we mapped the 36 ICI side effects and their associated CTCAE term(s) to FACIT items using the following process:

1. The following criteria was entered into the FACIT searchable library: ‘age = all’; ‘what are you searching for = individual items’; ‘search by = symptom’. This produced a list of 36 symptoms.
2. The ICI side effect and its CTCAE term(s) were then located within this list and relevant FACIT items were recorded. For each FACIT item, the item code and rating scale were recorded.

Mapping of side effects to the item libraries using the above process was conducted by JLK and IZ independently. Mapping results were then compared to calculate an inter-agreement rate for each item library.

#### Selecting the most appropriate items to measure each ICI side effect

Once items had been collated from the item libraries, JLK, IZ and CR selected items for each ICI side effect for inclusion in our ePRO system based on the following criteria.

i) Where available, we selected PRO-CTCAE items for an ICI side effect because a composite grading algorithm had been developed which produces a single composite numerical grade for each PRO-CTCAE symptomatic adverse event. This is based on its individual item scores for frequency, severity and/or interference [43]. This composite grade corresponds with the CTCAE, which is thought to improve clinician usability and interpretability, and link with immune-related adverse event (irAE) management guidelines. All relevant symptom attribute items for the PRO-CTCAE symptom term were included in the final item list.
ii) If PRO-CTCAE items were not available for a specific ICI side effect, items were selected from either the EORTC Item Library or the FACIT searchable library. As there is no direct relationship between the EORTC or FACIT item’s 5-point Likert response scale with a CTCAE grade, a custom algorithm will be created to associate responses with a CTCAE grade, which will be described in future studies.
iii) If we identified more than one appropriate EORTC or FACIT item for a specific ICI side effect, we selected the item based on the following criteria: face validity for assessing the ICI side effect as judged by a clinician, whether the item adequately represents the ICI side effect, and phrasing as a question rather than a statement to maintain consistency with included PRO-CTCAE items. If several items had similar face validity, all items were retained and presented to a group of patients and clinicians to determine the most appropriate item for inclusion [44].
iv) If no appropriate item existed, a new item was either adapted from an existing item’s stem or written according to established guidelines [44]. These items were intended to screen for side effects, rather than be used as an outcome measure that requires rigorous measurement properties for use in a clinical trial.

A provisional list of items for each side effect was then reviewed by a group of five patients and four oncologists to confirm clinical appropriateness. If several items had similar face validity, all items were presented with a proposed item highlighted. Items were discussed with the group. Based on group feedback, minor revisions to item wording were made.

## Results

### Mapping of ICI side effects to CTCAE terms

All 36 ICI side effects mapped to at least 1 CTCAE term. Eight side effects mapped to more than 1 CTCAE term- confusion or difficulty remembering things (CTCAE term: confusion, memory impairment), eye problems (eye pain, photophobia, uveitis), blood in stool (anal hemorrhage, colonic hemorrhage), joint problems (arthralgia, arthritis, joint range of motion decreased), fevers or chills (fevers, chills), unexplained bruising or bleeding from the nose or mouth (bruising, oral hemorrhage, epistaxis), swelling of the body (abdominal distension, oedema face, oedema limbs, generalized oedema), feeling upset or sad (depression, anxiety).

The inter-rater agreement rate for mapping of ICI side effects to CTCAE terms was 97% (35/36 side effects mapped to the same CTCAE terms).

### Mapping of ICI side effects/CTCAE terms to PRO-CTCAE terms

Twenty-three side effects and/or their CTCAE term(s) mapped to at least one PRO-CTCAE term. The side effect ‘Unexplained bruising and bleeding from the nose or mouth’ (CTCAE terms: bruising, oral hemorrhage, epistaxis) mapped to 2 PRO-CTCAE terms (‘bruising’, ‘nose bleed’).

Of the 24 PRO-CTCAE terms identified, 6 assessed all 3 attributes (frequency, severity and interference), 12 assessed 2 attributes, 4 assessed 1 attribute, and 2 assessed presence or absence only (rash and bruising). Of the 13 side effects that did not map to a PRO-CTCAE term, the majority were neurological side effects (seizures, drowsiness, loss of balance/ coordination, trouble walking, arm or leg weakness). The side effect ‘vision problems (e.g. double vision, loss of part of vision, blurred vision, change in color vision)’ was partially covered by the PRO-CTCAE term ‘blurred vision’, but other aspects such as double vision, loss of part of vision and change in color vision were not covered. Similarly, the side effect ‘eye problems (e.g. protrusion of the eye, sensitivity of the eyes to light, eye pain or red eye)’ was partially covered by the PRO-CTCAE terms ‘flashing lights’, but other aspects were not covered.

The inter-rater agreement rate on ICI side effects to PRO-CTCAE terms was 100% (36/36 side effects were mapped to the same PRO-CTCAE terms).

### Mapping of ICI side effects to EORTC items

35 side effects and their CTCAE term(s) mapped to at least 1 EORTC Item Library item. Blood in urine (CTCAE term: hematuria) was the only side effect that could not be mapped to an EORTC item. The side effect ‘rash’ was not included in the EORTC Item Library’s CTCAE classification. This is despite there being multiple CTCAE terms for dermatological (or skin/subcutaneous) conditions within the EORTC Item Library. Therefore, it was not possible to identify the EORTC item for ‘rash’ using our described process.

ICI side effects that mapped to the highest number of EORTC items were shortness of breath (CTCAE term: dyspnea) (36 items), confusion or difficulty remembering things (confusion, memory impairment) (26 items), feeling upset or sad (depression, anxiety) (21 items), diarrhea (diarrhea) (19 items), and nausea (nausea) (18 items). Side effects that mapped to the fewest EORTC items were seizures (seizure), loss of balance or coordination (ataxia), headache (headache), hearing impairment (hearing impaired), chest pain (chest pain- cardiac) and coughing up blood (bronchopulmonary hemorrhage) with 1 item each.

The inter-rater agreement rate on ICI side effects to EORTC Item Library items was 83% (30/36 ICI side effects mapped to the same EORTC items).

### Mapping of ICI side effects to FACIT items

Thirty-one ICI side effects and their CTCAE term(s) mapped to at least 1 FACIT item. Side effects that did not map to a FACIT item were drowsiness, trouble walking, rapid or irregular heart beat, coughing up blood and wheezing.

Side effects that mapped to the highest number of FACIT items were feeling upset or sad (44 items), confusion or difficulty remembering things (19 items), swelling of the body (face, limbs, abdomen) (18 items), unexplained bruising or bleeding from the nose or mouth (17 items), and shortness of breath (16 items). Side effects that mapped to the fewest FACIT items were muscle weakness, hearing impairment, dizziness or light headedness, blood in the stool, nausea, vomiting, and rash (1 item each).

The inter-rater agreement rate on ICI side effects to FACIT items was 75% (27/36 side effects were mapped to the same FACIT items).

An example of the mapping process to the CTCAE, PRO-CTCAE, EORTC Item Library and the FACIT searchable library for the side effect ‘diarrhea’ is shown in Table 1.

**Table 1:** Example of mapping process- diarrhea

Side effect	CTCAE term	PRO-CTCAE term	PRO-CTCAE items	EORTC CTCAE classification	EORTC items (item code)	FACIT symptom category	FACIT items (item code)
Diarrhea	Diarrhea	Diarrhea	In the last 7 days, how often did you have loose or watery stools (diarrhea/ diarrhea)?	Diarrhea	Have you had diarrhea? (Q20)	Bowel > diarrhea	I have diarrhea (diarrhea) (C5)
					Have you needed to get up at night to open your bowels? (Q217)		I have been bothered by diarrhea (diarrhea) (ICM1)
					Have you needed to take medication to control diarrhea? (Q242)		My problem with diarrhea (diarrhea) keeps/wakes me up at night (D6)
					What was the highest number of times you had to open your bowels in any 24 h period? (Q243)		I have abdominal cramps or discomfort due to my diarrhea (diarrhea) (D5)
					Did you have frequent bowel movements? (Q254)		I have to limit my activities because of diarrhea (diarrhea) (AA8)
					Did frequent bag changes occur during the day? (Q439)		I have to limit my social activity because of diarrhea (diarrhea) (D1)
					Did frequent bag changes occur during the night? (Q440)		I have to limit my physical activity because of diarrhea (diarrhea) (D2)
					Did frequent bowel movements occur during the day? (Q446)		I have to limit my sexual activity because of diarrhea (diarrhea) (D3)
					Did frequent bowel movements occur during the night? (Q447)		I am embarrassed by having diarrhea (diarrhea) (D4)
					Have you experienced having more than three bowel movements with liquid stools on a single day? (Q833)		I move my bowels more frequently than usual (ITF1)
					Have you experienced diarrhea so severe that you had to go to the toilet several times within a few hours? (Q834)		I am afraid to be far from a toilet (IT2)
					Have you had accidental leakage of liquid stool? (Q835)		
					Have you had loose stools during the night? (Q837)		
					Have you been distressed due to diarrhea? (Q838)		
					Have you had liquid or watery stools? (Q839)		
					Have you had abdominal discomfort due to diarrhea? (Q840)		
					Have you worried about being far from the toilet because of diarrhea? (Q841)		
					Have you had to limit your work or daily activities because of diarrhea? (Q842)		
					Have you had to limit your social activities because of diarrhea? (Q843)		

### Selection of the most appropriate items to monitor for each ICI side effect

Using the criteria outlined above, PRO-CTCAE items were selected for 23/36 side effects. For the remaining 13 side effects, the EORTC Item Library and FACIT searchable library were reviewed for possible items. Using the criteria outlined in the methods, JLK, IZ and CR reviewed all items and selected a proposed item. The proposed items were then reviewed by a group of patients and clinicians for acceptability, relevance, and clinical appropriateness. Item wording was carefully considered to ensure it accurately captured the patient experience and reflected the irAE the item was intended to capture. Comments from the group and how these informed amendments to individual items are shown in Supplementary Table 1. For example, for the side effect ‘muscle weakness’, four EORTC items and one FACIT item were reviewed. Three EORTC items were deemed either too generic (i.e. not relating specifically to muscle weakness e.g. EORTC item Q15 ‘have you felt weak’) or too specific (e.g. EORTC item Q726 ‘have you started things without difficulty but got weak as you went on’), while the FACIT item was phrased as a statement rather than a question (FACIT item HI12 ‘I feel weak all over’). The proposed item selected was EORTC item Q355 ‘have you had muscle weakness’. This item was discussed with a group of patients and clinicians who agreed that the question should specifically refer to ‘muscle’ weakness as opposed to ‘arm’ or ‘leg’ weakness and therefore selected Q355 as the final item.

The final 61-item list is shown in Table 2. Items were grouped according to body system for ease of patient administration. For 23 side effects, items from the PRO-CTCAE were selected. For the side effect ‘unexplained bruising or bleeding from the nose or mouth’, two PRO-CTCAE terms (‘bruising’, ‘nose bleed’) and their associated items were selected. For the remaining 13 side effects, items from the EORTC item library were selected for 11 side effects and items from the FACIT searchable library were selected for 1 side effect. For the side effect ‘eye problems’, the EORTC item Q378 and FACIT searchable library item Br6 were combined to create a new item. The wording of the recall period, which differed between item libraries, was also changed to ‘in the last 7 days’ to be consistent with the PRO-CTCAE given most items were from the PRO-CTCAE.

**Table 2:** Final customized item list

Side-effect	Item library (item code)	Item
Seizure	EORTC (Q222)	• In the last 7 days, have you had any seizures? (Response options: Not at all, a little, quite a bit, very much)
Confusion, difficulty remembering things	PRO-CTCAE (memory)	• In the last 7 days, what was the severity of your problems with memory at their worst? (Response options: none, mild, moderate, severe, very severe) • In the last 7 days, how much did problems with memory interfere with your usual or daily activities? (Response options: not at all, a little bit, somewhat, quite a bit, very much
Drowsiness	EORTC (Q358)	• In the last 7 days, have you felt drowsy? (Response options: Not at all, a little, quite a bit, very much)
Loss of balance or coordination	EORTC (Q241)	• In the last 7 days, have you had trouble with your balance or coordination? (Response options: Not at all, a little, quite a bit, very much)
Trouble walking	EORTC (based on Q5)	• In the last 7 days, have you had trouble walking? (Response options: Not at all, a little, quite a bit, very much)
Arm or leg weakness	EORTC (Q252)	• In the last 7 days, have you felt weak in your arms or legs? (Response options: Not at all, a little, quite a bit, very much)
Muscle weakness	EORTC (Q355)	• In the last 7 days, have you had muscle weakness? (Response options: Not at all, a little, quite a bit, very much)
Numbness or tingling in the hands or feet	PRO-CTCAE (numbness and tingling)	• In the last 7 days, what was the severity of your numbness or tingling in your hands or feet at its worst? (Response options: none, mild, moderate, severe, very severe) • In the last 7 days, how much did numbness or tingling in your hands or feet interfere with your usual or daily activities? (Response options: not at all, a little bit, somewhat, quite a bit, very much)
Headache	PRO-CTCAE (headache)	• In the last 7 days, how often did you have a headache? (Response options: never, rarely, occasionally, frequently, almost constantly) • In the last 7 days, what was the severity of your headache at its worst? (Response options: none, mild, moderate, severe, very severe) • In the last 7 days, how much did your headache interfere with your usual or daily activities? (Response options: not at all, a little bit, somewhat, quite a bit, very much)
Vision problems (for example- double vision, loss of part of your vision, blurred vision, or change in color vision)	EORTC (Q220)	• In the last 7 days, have you had trouble with your eyesight? (Response options: Not at all, a little, quite a bit, very much)
Eye problems (for example- protrusion of the eye, sensitivity of the eyes to light, eye pain, or red eye)	EORTC (Q378) and FACIT (Br6)	• In the last 7 days, have you had trouble with your eyes? (Response options: Not at all, a little, quite a bit, very much)
Hearing impairment	EORTC (Q144)	• In the last 7 days, have you had trouble with your hearing? (Response options: Not at all, a little, quite a bit, very much)
Chest pain	EORTC (Q396)	• In the last 7 days, have you had chest pain? (Response options: Not at all, a little, quite a bit, very much)
Rapid or irregular heart beat	PRO-CTCAE (heart palpitations)	• In the last 7 days, how often did you feel a pounding or racing heart beat (palpitations)? (Response options: never, rarely, occasionally, frequently, almost constantly) • In the last 7 days, what was the severity of your pounding or racing heart beat (palpitations) at its worst? (Response options: none, mild, moderate, severe, very severe)
Light-headedness/ dizziness	PRO-CTCAE (dizziness)	• In the last 7 days, what was the severity of your dizziness at its worst? (Response options: none, mild, moderate, severe, very severe) • In the last 7 days, how much did dizziness interfere with your usual activities? (Response options: not at all, a little bit, somewhat, quite a bit, very much)
Shortness of breath	PRO-CTCAE (shortness of breath)	• In the last 7 days, what was the severity of your shortness of breath at its worst? (Response options: none, mild, moderate, severe, very severe) • In the last 7 days, how much did your shortness of breath interfere with your usual or daily activities? (Response options: not at all, a little bit, somewhat, quite a bit, very much)
Cough	PRO-CTCAE (cough)	• In the last 7 days, what was the severity of your cough at its worst? (Response options: none, mild, moderate, severe, very severe) • In the last 7 days, how much did cough interfere with your usual or daily activities? (Response options: not at all, a little bit, somewhat, quite a bit, very much)
Coughing up blood	EORTC (Q470)	• In the last 7 days, have you coughed up blood? (Response options: Not at all, a little, quite a bit, very much)
Wheezing	PRO-CTCAE (wheezing)	• In the last 7 days, what was the severity of your wheezing (whistling noise in the chest with breathing) at its worst? (Response options: none, mild, moderate, severe, very severe)
Abdominal pain	PRO-CTCAE (abdominal pain)	• In the last 7 days, how often did you have pain in the abdomen (belly area)? (Response options: never, rarely, occasionally, frequently, almost constantly) • In the last 7 days, what was the severity of your pain in your abdomen (belly area) at its worst? (Response options: none, mild, moderate, severe, very severe) • In the last 7 days, how much did pain in the abdomen (belly area) interfere with your usual or daily activities? (Response options: not at all, a little bit, somewhat, quite a bit, very much)
Diarrhea	PRO-CTCAE (diarrhea)	• In the last 7 days, how often did you have loose or watery stools (diarrhea)? (Response options: never, rarely, occasionally, frequently, almost constantly)
Blood in stool	EORTC (Q55)	• In the last 7 days, have you had blood in your stools (bowel movements)? (Response options: Not at all, a little, quite a bit, very much)
Nausea	PRO-CTCAE (nausea)	• In the last 7 days, how often did you have nausea? (Response options: never, rarely, occasionally, frequently, almost constantly) • In the last 7 days, what was the severity of your nausea at its worst? (Response options: none, mild, moderate, severe, very severe)
Vomiting	PRO-CTCAE (vomiting)	• In the last 7 days, how often did you have vomiting? (Response options: never, rarely, occasionally, frequently, almost constantly) • In the last 7 days, what was the severity of your vomiting at its worst? (Response options: none, mild, moderate, severe, very severe)
Difficulty swallowing	PRO-CTCAE (difficulty swallowing)	• In the last 7 days, what was the severity of your difficulty swallowing at its worst? (Response options: none, mild, moderate, severe, very severe)
Blood in the urine	FACIT (RCC2)	• In the last 7 days, have you had blood in your urine? (Response options: Not at all, a little, quite a bit, very much)
Going to the toilet more often than normal to pass urine OR drinking more fluids than normal	PRO-CTCAE (urinary frequency)	• In the last 7 days, were there times when you had to urinate frequently? (Response options: never, rarely, occasionally, frequently, almost constantly) • In the last 7 days, how much did frequent urination interfere with your usual or daily activities? (Response options: not at all, a little bit, somewhat, quite a bit, very much)
Rash	PRO-CTCAE (rash)	• In the last 7 days, have you had a rash? (Response options: Presence/ absence)
Itching	PRO-CTCAE (itching)	• In the last 7 days, what was the severity of your itchy skin at its worst? (Response options: none, mild, moderate, severe, very severe)
Mouth ulcers	PRO-CTCAE (mouth/ throat sores)	• In the last 7 days, what was the severity of your mouth/ throat sores at its worst? (Response options: none, mild, moderate, severe, very severe) • In the last 7 days, how much did mouth or throat sores interfere with your usual or daily activities? (Response options: not at all, a little bit, somewhat, quite a bit, very much)
Joint problems (for example- swelling, pain, stiffness)	PRO-CTCAE (joint pain)	• In the last 7 days, how often did you have aching joints (such as elbows, knees, shoulders)? (Response options: never, rarely, occasionally, frequently, almost constantly) • In the last 7 days, what was the severity of your aching joints (such as elbows, knees, shoulders) at its worst? (Response options: none, mild, moderate, severe, very severe) • In the last 7 days, how much did aching joints (such as elbows, knees, shoulders) interfere with your usual or daily activities? (Response options: not at all, a little bit, somewhat, quite a bit, very much)
Muscle pain/ stiffness	PRO-CTCAE (muscle pain)	• In the last 7 days, how often did you have aching muscles? (Response options: never, rarely, occasionally, frequently, almost constantly) • In the last 7 days, what was the severity of your aching muscles at its worst? (Response options: none, mild, moderate, severe, very severe) • In the last 7 days, how much did aching muscles interfere with your usual or daily activities? (Response options: not at all, a little bit, somewhat, quite a bit, very much)
Fevers or chills	PRO-CTCAE (chills)	• In the last 7 days, how often did you have shivering or shaking chills? (Response options: never, rarely, occasionally, frequently, almost constantly) • In the last 7 days, what was the severity of your shivering or shaking chills at its worst? (Response options: none, mild, moderate, severe, very severe)
Unexplained bruising or bleeding from the nose or mouth	PRO-CTCAE (bruising)	• In the last 7 days, did you bruise easily (black and blue marks)? (Response options: yes/ no)
	PRO-CTCAE (nose bleed)	• In the last 7 days, how often did you have nosebleeds? (Response options: never, rarely, occasionally, frequently, almost constantly) • In the last 7 days, what was the severity of your nosebleeds at their worst? (Response options: none, mild, moderate, severe, very severe)
Swelling of the body (face, limbs, abdomen)	PRO-CTCAE (swelling)	• In the last 7 days, how often did you have arm or leg swelling? (Response options: never, rarely, occasionally, frequently, almost constantly) • In the last 7 days, what was the severity of your arm or leg swelling at its worst? (Response options: none, mild, moderate, severe, very severe) • In the last 7 days, how much did arm or leg swelling interfere with your usual or daily activities? (Response options: not at all, a little bit, somewhat, quite a bit, very much)
Feeling upset or sad	PRO-CTCAE (sad)	• In the last 7 days, how often did you have sad or unhappy feelings? (Response options: never, rarely, occasionally, frequently, almost constantly) • In the last 7 days, what was the severity of your sad or unhappy feelings at their worst? (Response options: none, mild, moderate, severe, very severe) • In the last 7 days, how much did sad or unhappy feelings interfere with your usual or daily activities? (Response options: not at all, a little bit, somewhat, quite a bit, very much)

## Discussion

Advances in cancer therapy have resulted in the increasing use of novel therapies with novel side effect profiles as part of routine clinical care. However, existing static PROMs have not consistently kept pace with these therapeutic developments, resulting in inadequate coverage of the side effects of novel therapies. Pragmatic patient-centered approaches are therefore needed to ensure PROMs remain agile and fit-for-purpose for measuring side effects of novel therapies. The advent of item libraries has facilitated a more flexible approach to measuring novel side effects through the creation of customized item lists, rather than necessitating the lengthy development of an entirely new PROM.

This work specifically addresses the need to improve the clinical utility of PROMs used in routine clinical care, where one of the key contemporary uses of PROs is ePRO symptom monitoring, and where one of the key challenges is how to create customized item lists to monitor for treatment-related side effects. Currently, there is minimal guidance on how to undertake this process rigorously.

This work addresses this challenge by proposing a systematic process for mapping side effects relevant to a specific treatment to validated items from three major item libraries, using ICI side effects as a test case for this proposed approach. It highlights the practical challenges of doing this and outlines ways in which item libraries can be improved/ adapted to make them fit for this purpose.

### Mapping of side effects to CTCAE terms

We considered mapping side effects to the CTCAE a necessary step to ensure the terminology used for each side effect was aligned with an internationally recognized framework (the CTCAE). This was felt to improve the accuracy of the mapping to the item libraries, particularly those that allow searching by CTCAE term (e.g. EORTC Item Library). However, not all side effects identified in our Delphi survey aligned with a CTCAE term. For example, there were no suitable CTCAE terms for ICI side effects of joint swelling, double vision, or change in color vision. This lack of CTCAE term highlights gaps within the CTCAE for certain patient-reported side effects that could be addressed in future work.

### Mapping of side effects to item libraries

Some ICI side effects mapped to multiple items within an item library or multiple items across different item libraries. Currently, there is limited guidance about how to best select items. Piccinin et al. suggest that when creating customized item lists for use in clinical trials, items should be selected based on the item’s face validity for the study’s aims/ research questions and be suitable for the patient population under investigation [42]. This could also be applied to the routine care context. We applied Piccinin et al.’s [42] guidance in designing our process for selecting items within and across item libraries. We established clear item selection criteria prior to selecting items and the clinical relevance of our chosen items was confirmed by both consumers and clinicians. Item libraries could also consider recommending a preferred item for measuring a specific side effect to aid this process. Work is currently underway within the EORTC Quality of Life Group to recommend a preferred item for specific side effects within the EORTC Item Library.

Not all side effects map to items within a single item library, necessitating sourcing items from more than one item library to ensure adequate coverage of side effects. However, variation in wording of recall period (‘in the last 7 days’, ‘in the past 7 days’, ‘during the past week’), reporting requirements (worst symptoms in the past 7 days versus an average of symptoms experienced in the past 7 days), phrasing of items (positive vs. negative phrasing, ‘trouble with’ vs. ‘difficulties’ vs ‘problems’), tense (‘had’ vs. ‘have’) and response scales (4 point vs. 5 point, different responses within each scale) make it difficult to include items from different item libraries within a single item list. Indeed, even within a single library, items may have different stems (‘troubles with’, ‘difficulties with’, ‘problems with’) and tenses (‘had’, ‘have’). Item libraries could consider having a process to review their current content to ensure consistency of wording, although it is recognized that harmonization across different item libraries may not be achievable.

Currently, there are no clear guidelines on what to do if this situation arises. The need to source items from multiple item libraries to provide adequate coverage of relevant side effects highlights the need for item libraries to be regularly updated to include emerging toxicities/ side effects of novel therapies. For example, 13 side effects did not map to the PRO-CTCAE, with the majority of these representing neurological side effects, indicating areas where new PRO-CTCAE items could be developed. Item libraries are typically populated from developed PROMs and rely on the validation of new PROMs to add new items to the library. Developing new methods for developing new items in a more expedited way in response to new classes of drugs outside of traditional PROM development may be helpful. This will not only minimize the need for item selection from more than one item library, but more importantly, will ensure side effects can be consistently monitored in both clinical trials and routine care. Should items from multiple item libraries be required, these could be presented in separate sections of the side effect survey to minimize cognitive burden. Furthermore, if items require revision, items with similar wording (e.g. a similar stem) could also be presented together to minimize cognitive response burden.

### Using responses to inform clinical decision making

Once the side effects for monitoring have been identified, questions to establish the frequency, severity and degree of interference for each side effect are needed to determine the overall severity of the side effect. This then allows clinicians to arrange appropriate investigations and management.

However, the scoring of items from item libraries may also not clearly link with established methods of grading side effects such as the CTCAE. Whilst the PRO-CTCAE has a composite grading algorithm which produces a grade that is analogous to the grading scale employed by the CTCAE [43], this does not always completely align with a CTCAE grade. For example, for rash, calculation of a CTCAE grade requires an assessment of both its extent (in terms of body surface area affected), presence or absence of associated symptoms (such as pruritus, burning or tightness) as well as its impact on instrumental and self-care activities of daily living. However, not all aspects are assessed within the PRO-CTCAE, which only asks about the presence of absence of the rash. Clinicians may also be better positioned to assess certain attributes such as extent. The EORTC is in the process of linking its five-point response scales to CTCAE grades [45]. To our knowledge, similar work has not been planned for FACIT items. The absence of a clear linkage between responses to items from item libraries and the CTCAE may limit their usability for remote symptom monitoring. Item responses need to ideally align with the CTCAE to maximize clinician interpretability and usability given that many existing investigation and management algorithms are based on a CTCAE grade.

One possible alternative is to use plain language versions of the CTCAE, such as the adapted REQUITE questionnaire [46, 47]. Whilst this approach provides a direct linkage between patient responses and a CTCAE grade and was preferred by patients compared to the PRO-CTCAE in a small single site study of patients with lung cancer [47], some patients have reported it is more complex to understand [47] and therefore requires further validation with a broader patient group.

This work has several strengths. Our study represents one of the first attempts to describe in detail the exact process of selecting PRO items from three available item libraries to create a customized item list for measuring side effects of a novel therapy. This process can be used by others selecting PRO items for inclusion in remote monitoring systems. While others have created customized lists from item libraries, these have typically selected items from a single item library, including the PRO-CTCAE [29–33], EORTC Item Library [34] and the FACIT searchable library [35]. This study provides the first detailed, pragmatic description of how to select items across multiple item libraries to best characterize a particular side effect. We developed clear criteria for selecting items which could be adapted for customized lists for any purpose and included patient and clinician input into the final list. Møller et al. developed an item set for acute treatment toxicities from pelvic online magnetic resonance guided radiotherapy [48] using items from the PRO-CTCAE and the EORTC Item Library. Where items were available in both libraries, the authors stated that the wording of the item influenced the item selected but the exact process for doing this was not described. Our approach could represent a ‘minimum standard’ for selecting items to monitor for side effects in routine care, balancing the rigor of using PROMs in the context of clinical trials and current clinical practice where PROMs are often used ‘off the shelf’ and may lack content and face validity.

Limitations of this study include the fact that the process for mapping side effects to item library items was created by the study team, as there are no guidelines for how to do this. However, it was informed by existing guidelines, including the EORTC Item Library User Guidelines [40], Piccinin et al. [42], and the mapping process undertaken by Gilbert et al. [41], and supported by co-authors representing the three item libraries. Furthermore, given the high levels of inter-rater reliability demonstrated, this method could be considered feasible for clinicians wanting to replicate the process for other novel therapies.

## Conclusion

We developed a pragmatic approach to creating customized item lists from existing major item libraries for measuring the side effects of novel therapies using the example of ICIs. This can be applied to the creation of customized item lists for use in routine clinical care, such as for ePRO symptom monitoring. This process has highlighted the challenges of using item libraries and identified priorities for future methodological work to improve their usability. This work needs to be prioritized to meet the contemporary needs of those receiving and delivering novel therapies.

## Electronic supplementary material

Below is the link to the electronic supplementary material.

Supplementary Material 1: Supplementary table 1: EORTC and FACIT items- comments from patient and clinician group and subsequent amendments.

## Data Availability

De-identified participant data underlying the results reported in this article will be shared. Data will be available immediately following publication with no end date. Investigators who propose use of the data that has been approved by an independent review committee will be granted access to the data to achieve the aims of the approved proposal. Proposals should be directed to Julia.Lai-Kwon@petermac.org.
